# POP-Q Versus Upright MRI Distance Measurements: A Prospective Study in Patients with POP

**DOI:** 10.1007/s00192-024-05802-7

**Published:** 2024-05-14

**Authors:** Annemarie van der Steen, Kim Y. Jochem, Esther C. J. Consten, Frank F. J. Simonis, Anique T. M. Grob

**Affiliations:** 1 Multi Modality Medical Imaging (M3I), TechMed Centre, University of Twente, Drienerlolaan 5, 7522 NB Enschede, The Netherlands; 2 Department of Gynecology, Ziekenhuisgroep Twente, Hengelo, The Netherlands; 3 Department of Surgery, Meander Medisch Centrum, Amersfoort, The Netherlands; 4 Department of Surgery, University Medical Center Groningen, Groningen, The Netherlands; 5 Magnetic Detection and Imaging (MD&I), TechMed Centre, University of Twente, Enschede, The Netherlands

**Keywords:** Correlation, Magnetic resonance imaging, Pelvic organ prolapse, POP-Q, Quantification, Upright

## Abstract

**Introduction and Hypothesis:**

The gold standard for quantifying pelvic organ prolapse is the pelvic organ prolapse quantification (POP-Q) system; however, upright magnetic resonance imaging (MRI) is a promising new method. The objective of this study was to determine the correlation between POP-Q and MRI measurements of the bladder and cervix.

**Methods:**

This prospective study included patients with prolapse in whom POP-Q points Aa or Ba and C were measured as standard care. MRI scans were performed in an upright position, and the distances of the lowest points of the bladder and cervix to the Pelvic Inclination Correction System (PICS) were calculated. Correlations between POP-Q and MRI-PICS measurements were determined using the Pearson correlation coefficient for normally distributed data and the Spearman’s rank correlation coefficient for non-normally distributed data.

**Results:**

A total of 63 patients were suitable for analysis. There was a moderate positive correlation between the POP-Q and MRI-PICS measurements for bladder (*r*(61) = 0.480, *r* < 0.001) and uterus (*r*(61) = 0.527, *p* < 0.001). Measurement differences between POP-Q and MRI-PICS of the bladder and uterus vary from −3.2 cm to 7.1 cm, and from −2.1 cm to 8.5 cm respectively. In 71.4% of patients more descent was seen on upright MRI than with POP-Q measurement for both bladder and uterus. For patients with similar POP-Q measurements, a high variation in MRI measurements of the bladder and uterus was found.

**Conclusion:**

Despite a moderate positive correlation, upright MRI shows a larger POP extent in 71.4% of the patients than POP-Q. A high variation in MRI measurements for patients with the same POP-Q measurement was seen.

## Introduction

Pelvic organ prolapse (POP) is a common condition in women, with a reported prevalence of symptomatic POP of 11.4% [1]. POP is defined as a downward displacement of the anterior vaginal wall, the uterus or vaginal vault, the posterior vaginal wall, or a combination of any of them [2]. Correct diagnosis of POP is important as it influences treatment decisions, such as pelvic floor physiotherapy, pessary treatment, and surgical treatment [3].

The Pelvic Organ Prolapse Quantification (POP-Q) system is the gold standard for quantifying POP [4], and is the recommended system by among others the International Continence Society, American College of Obstetricians and Gynecologists, and the Royal College of Obstetricians and Gynaecologists [5–7]. However, this method has several limitations. First, POP-Q examination is usually performed with the patient in a dorsal lithotomy position, whereas symptoms are mostly experienced in an upright position. Previous research confirmed that intraoperative POP assessment with cervical traction revealed a larger extent of POP than was established at preoperative evaluation [8–10]. Second, the measurements are performed using the hymen as a reference point. The hymen is not a fixed point and will move downward with Valsalva along with the prolapsed organs [11]. Finally, the Valsalva maneuver itself, which is used for POP-Q assessment, is sometimes difficult to perform for patients. All three limitations of the POP-Q assessment can lead to underestimation of the extent of POP and can explain the poor association between POP-Q stage and severity of symptoms [12, 13]. We therefore hypothesize that POP-Q examination might not be the most reliable method of quantifying POP.

Imaging techniques, such as ultrasound and magnetic resonance imaging (MRI), have been investigated for quantifying POP. Because MRI has a good spatial resolution, large field of view, and good soft-tissue contrast, it is especially suitable for providing information about pelvic organs and supportive structures [14]. Assessment of the extent of POP based on dynamic MRI shows an outstanding inter- and intra-observer reliability, but POP quantification on supine MRI had a poor-to-moderate correlation with physical examination and POP symptoms [15–17]. In addition, Grob et al. concluded that MRI scanning of patients with POP stage ≥ 2 shows a significantly larger extent of the prolapse in upright rest than during a supine straining position. This indicates the need to investigate POP quantification with upright MRI and its correlation with POP-Q assessment [18]. We hypothesize that upright MRI measurements can provide a more reliable POP quantification than the POP-Q system in a dorsal lithotomy position. Therefore, in the future, upright MRI may provide a better understanding of patients’ symptoms and a better preparation for surgical treatment.

The aim of this prospective research is to establish the correlation between the distance measurements of the bladder and uterus to the pelvic inclination correction system (PICS) plane in upright MRI, and the POP-Q measurements of the anterior vaginal wall and cervix in patients with POP.

## Materials and Methods

### Population

Magnetic resonance imaging scans and POP-Q measurements of patients with POP recruited for two different POP studies were included in this study. Consecutive patients were recruited between 2021 and 2023 from the gynecology department of the Ziekenhuisgroep Twente hospital in Almelo and Hengelo, the Netherlands. Both studies were approved by the medical ethics committee (NL74061.091.20 and NL79717.091.21) and all patients gave written informed consent. Patients were not involved in the designing or conducting of the research. All women were 18 years or older and had a minimum stage 2 prolapse of the anterior vaginal wall or uterus, and had not undergone previous POP surgery. As prolapse of the posterior compartment is difficult to visualize on MRI without using rectal contrast, patients with primarily a posterior vaginal wall prolapse were not included in the study. Patients were excluded if they were not able to stand for 20 min without assistance, were not eligible to undergo an MRI scan in response to an MRI safety checklist, or had a jeans size ≥ 52 (EU) or 22 (US), because of the limited coil circumference.

### MRI Examination

Magnetic resonance scans of the women in an upright position and the pelvis at rest were acquired. The participants were not allowed to drink for 1 h before the scan and had to empty their bladder within 15 min before the scan. A tiltable 0.25 T MR scanner (G-Scan Brio; Esaote S.p.A., Genoa, Italy) was used for MRI acquisition, with a dedicated multichannel spine coil. A 3D balanced steady-state free precession sequence was acquired in an upright patient position (TE/TR: 4/8 ms, flip angle: 60°, reconstructed resolution: 0.49 × 0.49 × 0.49 mm^3^, FOV: 250 × 250 × 122 mm^3^ or 250 × 250 × 160 mm^3^, acquisition matrix 124 × 124 × 100, number signal averages 3, scan time: ± 5 min).

### POP Measurements

In all women a POP-Q measurement in a dorsal lithotomy position, and under maximum Valsalva had been performed as part of standard care by one of the urogynecologists. Points “Aa” or “Ba” (whichever showed the highest value) were used for the quantification of the anterior vaginal wall (from now on called “bladder”) prolapse, and “C” was used for the uterus prolapse.

To measure the extent of POP with MRI several reference lines have been proposed over time [19]. In 2017 Reiner et al. introduced the Pelvic Inclination Correction System, which has several advantages over the other reference lines, including the possibility of evaluating POP in three dimensions (3D) [20]. The method was validated for upright MRI, using a 29° PICS angle, by Morsinkhof et al. [21]. The MRI-PICS plane was determined by manually selecting the right ischial spine, the left ischial spine, the inferior pubic point, and the sacrococcygeal joint, as described by Reiner et al. [20]. Following the selection of the PICS plane the lowest points of the bladder and uterus were annotated manually on sagittal MRI slices using a 3D slicer (v.5.0.2) [22]. The rectum was not taken into account in these measurements as it was not possible to visualize the lowest point without the use of rectal contrast. After annotation, the coordinates of the points were exported to MATLAB (R2022a; MathWorks Inc., Natick, MA, USA). In MATLAB the perpendicular distances of the bladder and uterus to the PICS plane were calculated, where the negative values represent a position cranial from the PICS plane and the positive values a point caudal from the PICS plane. All MRI measurements were performed by one of the researchers (KJ) and when in doubt double checked by a second researcher (AS). The researchers were blinded to the POP-Q measurements during the performance of the MRI measurements.

### Statistical Analysis

Statistical analysis was performed using IBM SPSS Statistics (version 28.0.1.0, SPSS Inc., Chicago, IL, USA). Normality of the data was assessed by means of the Shapiro–Wilk test. The correlation between the POP-Q measurements and the MRI-PICS measurements were determined by the Pearson correlation coefficient for the normally distributed data and by the Spearman’s rank correlation coefficient for the non-normally distributed data. A statistical significance level of 5% was used for both correlations.

## Results

**Fig. 1:**
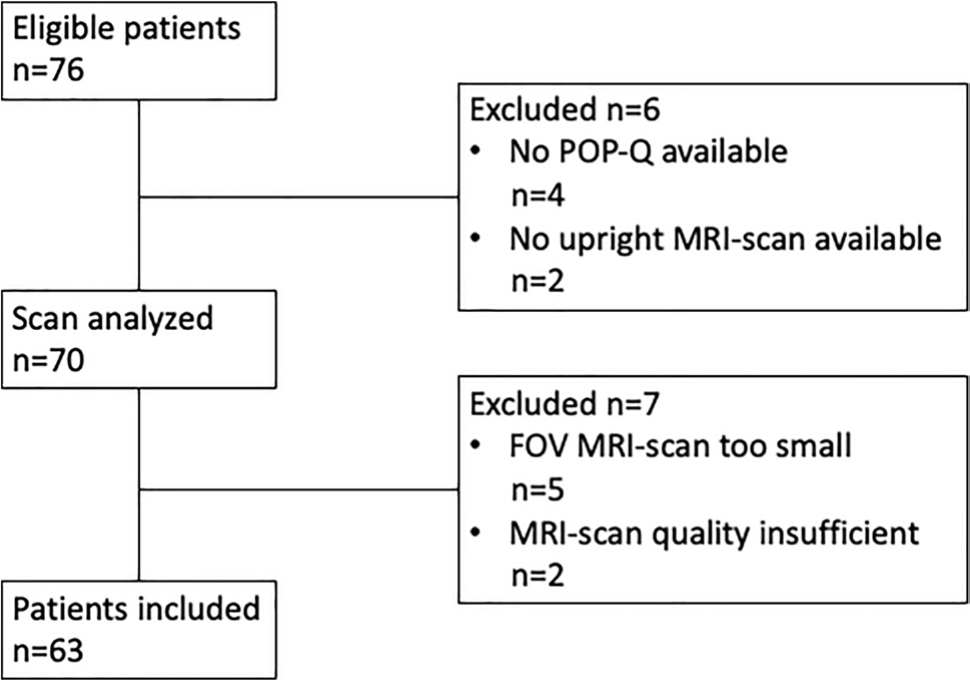
Flowchart of the patient inclusion and exclusion process. *POP-Q* Pelvic Organ Prolapse Quantification, *MRI* magnetic resonance imaging, *FOV* field of view

Out of a total of 76 eligible patients, 13 patients were excluded because no POP-Q was available (*n* = 4), no upright MRI scan was available because of the patients fainting in an upright position (*n* = 2), because the field of view (FOV) of the MRI scan was too small (*n* = 5), or because the MRI scan had insufficient image quality (*n* = 2), leaving 63 patients for analysis (Fig. 1).

Table 1 shows the baseline characteristics of the study population with the POP-Q stage of the bladder and uterus.

**Table 1:** Baseline characteristics of the study population (*n* = 63)

Age (years)		60.4 ± 9.9
BMI		26.9 ± 3.9
Parity^a^		2 (1–6)
POP-Q stage bladder^b^	Stage 1	3 (4.8%)
	Stage 2	30 (47.6%)
	Stage 3	29 (46.0%)
	Stage 4	1 (1.6%)
POP-Q stage uterus^b^	Stage 1	38 (60.3%)
	Stage 2	15 (23.8%)
	Stage 3	10 (15.9%)
	Stage 4	0 (0%)

Data presented as mean ± SD, median (range), or number of patients (percentage)

*BMI* body mass index, *POP-Q* Pelvic Organ Prolapse Quantification

The Shapiro–Wilk test showed that the POP-Q (W = 0.966, *p* = 0.077) and MRI-PICS (W = 0.965, *p* = 0.074) measurements for the bladder can be assumed to be normally distributed. For the uterus the POP-Q (W = 0.962, *p* = 0.049) and MRI-PICS (W = 0.934, *p* = 0.002) measurements were significantly different from normally distributed data.

The Pearson correlation coefficient indicated a moderate positive correlation between the POP-Q bladder measurements and the MRI-PICS bladder measurements (*r*(61) = 0.480, *p* < 0.001). The Spearman’s rank correlation coefficient indicated a moderate positive correlation between the POP-Q uterus measurements and the MRI-PICS uterus measurements (*r*(61) = 0.527, *p* < 0.001).

### Bladder

**Fig. 2:**
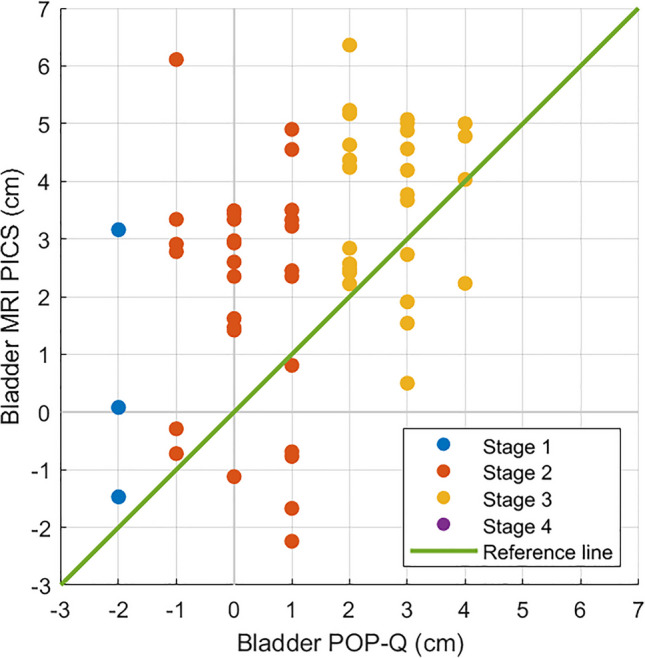
Scatterplot of the Pelvic Organ Prolapse Quantification (*POP-Q*) and magnetic resonance imaging-Pelvic Inclination Correction system (*MRI-PICS*) measurements of the bladder. POP-Q (x-axis) and MRI-PICS (y-axis) measurements of the bladder showing that MRI-PICS measurements are often larger. Data points are color labeled by POP-Q stage. The *green line* represents the reference line at which POP-Q and MRI-PICS values are equal

**Fig. 3:**
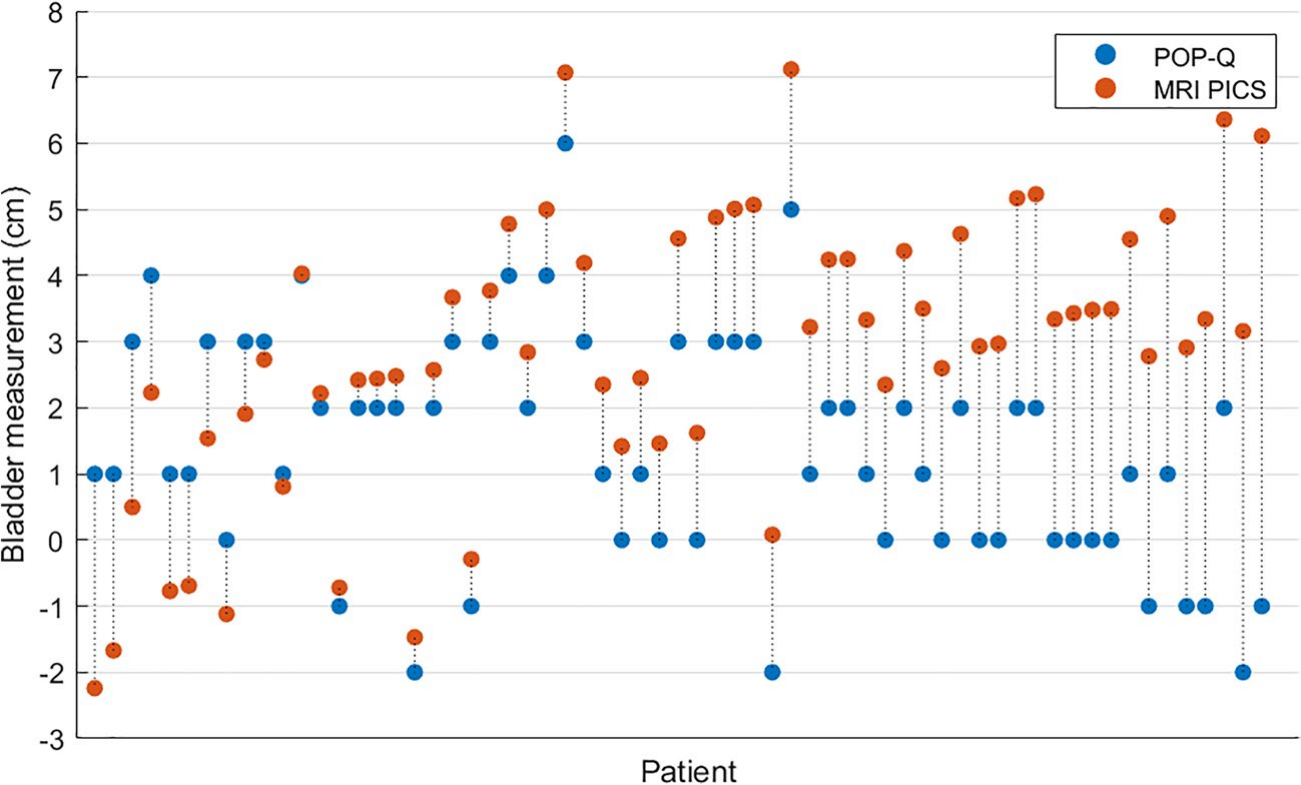
Scatterplot of the Pelvic Organ Prolapse Quantification (*POP-Q*) and magnetic resonance imaging-Pelvic Inclination Correction system (*MRI-PICS*) measurements of the bladder plotted individually per patient. The *red datapoints* show the MRI-PICS measurements per patient and are connected by the *dotted line* to the corresponding *blue datapoints* representing the POP-Q measurements. All patients are sorted by measurement difference

Figure 2 shows a scatterplot of the POP-Q and MRI-PICS measurements of the bladder, indicating the variety of MRI-PICS measurements per POP-Q outcome. For instance, when considering the bladder at POP-Q measurement + 1, a large variation in MRI-PICS measurements was found, with differences up to 7.1 cm between patients. Figure 3 illustrates the discrepancy between POP-Q and MRI-PICS measurements per patient, identifying three groups: patients in whom the MRI-PICS measurement is smaller (difference < −0.5 cm) than the POP-Q measurement (*n* = 9 [14.3%]); patients in whom the measurements were almost identical (absolute difference < 0.5 cm; *n* = 9 [14.3%]); and patients in whom the MRI-PICS measurement was larger (difference > 0.5 cm) than the POP-Q measurement (*n* = 45 [71.4%]). The measurement differences between the POP-Q and the MRI-PICS of the bladder vary from −3.2 cm to 7.1 cm.

### Uterus

**Fig. 4:**
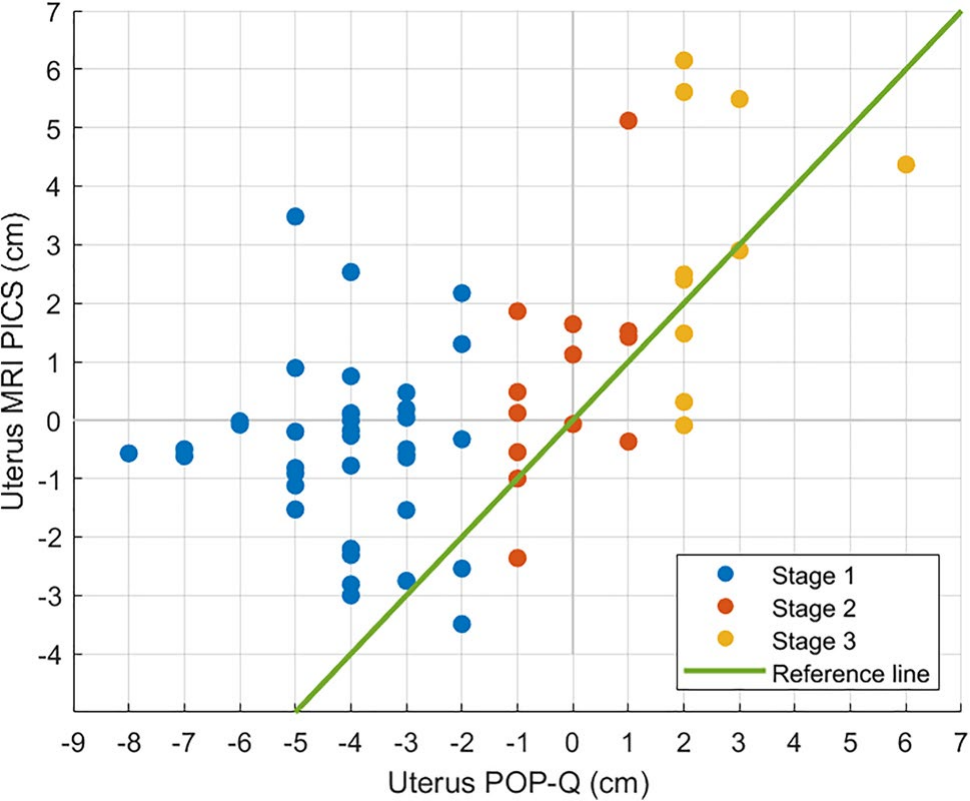
Scatterplot of the Pelvic Organ Prolapse Quantification (*POP-Q*) and magnetic resonance imaging-Pelvic Inclination Correction system (*MRI-PICS*) measurements of the uterus. POP-Q (x-axis) and MRI-PICS (y-axis) measurements of the uterus showing that MRI-PICS measurements are often larger. Data points are color labeled by POP-Q stage. The *green line* represents the reference line at which POP-Q and MRI-PICS values are equal

**Fig. 5:**
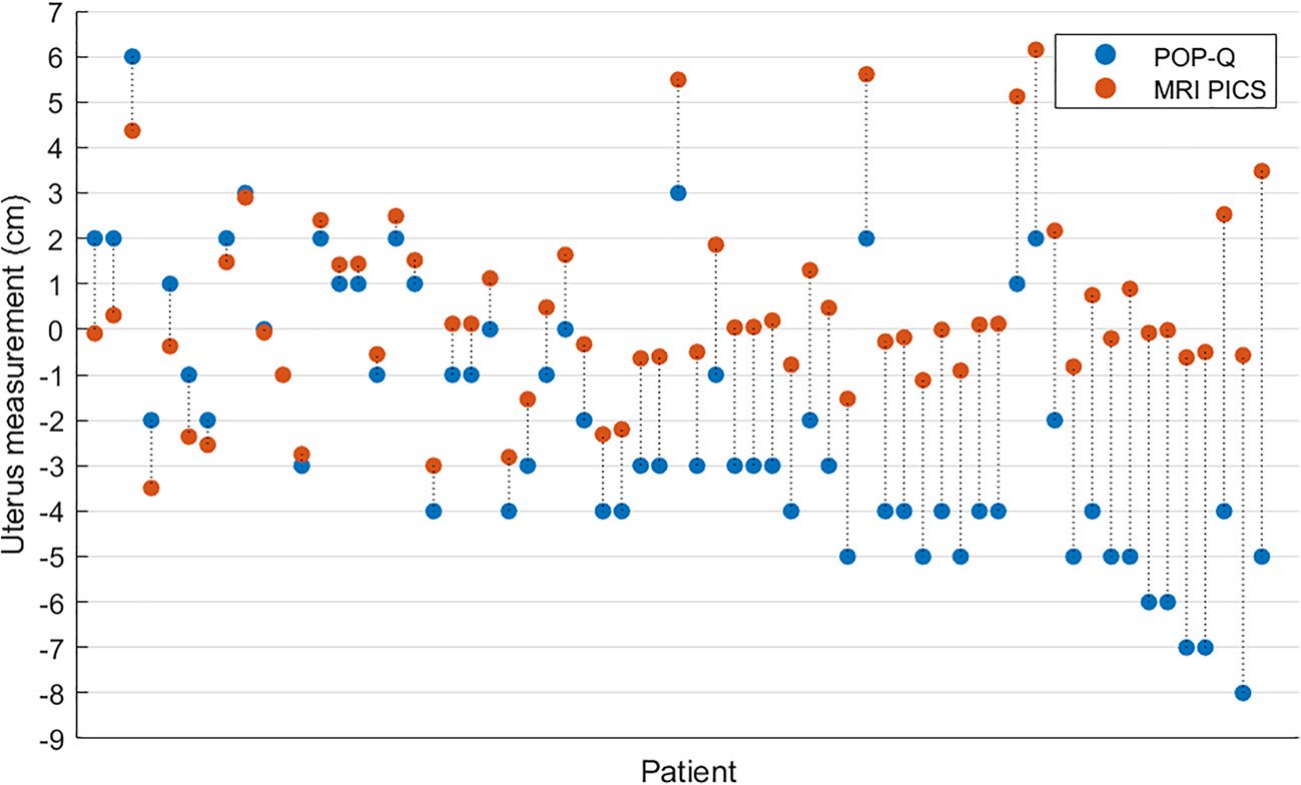
Scatterplot of the Pelvic Organ Prolapse Quantification (*POP-Q*) and magnetic resonance imaging-Pelvic Inclination Correction system (*MRI-PICS*) measurements of the uterus plotted individually per patient. The *red datapoints* show the MRI-PICS measurements per patient and are connected by the *dotted line* to the corresponding *blue datapoints* representing the POP-Q measurements. All patients are sorted by measurement difference

Figure 4 shows a scatterplot of the POP-Q and MRI-PICS measurements of the uterus, indicating the variety of MRI-PICS measures per POP-Q outcome. For instance, when considering the uterus at POP-Q measurement −4, a large variation in MRI-PICS measurements was found, with differences up to 5.5 cm between patients. Figure 5 illustrates the discrepancy between POP-Q and MRI-PICS measurements per patient, identifying three groups: patients in whom the MRI-PICS measurement is smaller (difference < −0.5 cm) than the POP-Q measurement (*n* = 6 [9.5%]); patients in whom the measurements were almost identical (absolute difference < 0.5 cm (*n* = 12 [19.0%]); and patients in whom the MRI-PICS measurement was larger (difference > 0.5 cm) than the POP-Q measurement (*n* = 45 [71.4%]). Of the patients with a stage 1 uterus prolapse specifically, 92% show greater descent of the uterus of 3.6 ± 2.2 cm on MRI-PICS compared with POP-Q. The measurement differences between the POP-Q and the MRI-PICS of the uterus vary from −2.1 cm to 8.5 cm.

## Discussion

### Main Findings

This prospective study showed a significant moderate positive correlation between the POP-Q and MRI-PICS measurements for the bladder and uterus in patients with POP. Furthermore, MRI-PICS measurements varied widely in patients with the same POP-Q values, and in 71.4% of the bladder and uterus measurements, the MRI-PICS measurements showed greater organ descent than the POP-Q measurements.

### Strengths and Limitations

The most important strength of this study is the use of upright instead of supine MRI. The upright rest position gives the best reflection of the natural organ position in patients with POP during the day. Another strong point in our study is that all the MRI-PICS measurements were performed in 3D, by one observer, following a dedicated protocol, reducing possible observer variation [20, 21].

Our study has several limitations. The widely varying MRI-PICS measurements for patients with the same POP-Q measurement could be explained by inaccuracies in the POP-Q measurements. As is known from literature, under controlled circumstances POP-Q has a high interobserver correlation [23]. However, in general clinical practice, as in our hospital, there is great variation in the execution of the POP-Q measurements. For example, not all patients are able to perform the Valsalva maneuver properly, specula are not used by all gynecologists, nor are rulers. This variation may have influenced our study results; however this effect, if at all present, would be minor and the variation reflects clinical practice [11, 24, 25]. As was mentioned earlier, we do not suspect the MRI-PICS measurements to be inaccurate, owing to the protocol that was performed.

Second, making a direct comparison between POP-Q and MRI-PICS is difficult because the hymen, which is the reference point for the POP-Q, was not visible on the MRI scans. We hypothesize that the position of the PICS plane might be more cranial than the hymen, which can account for the greater extent of POP measured with MRI-PICS than with POP-Q in the majority of the patients. However, the hymen as a reference point is not as fixed as the PICS plane and therefore comparing absolute values between POP-Q and MRI-PICS is not useful. Besides, this would not have influenced the currently found moderate positive correlation and cannot explain the large variety of measurements for the same POP-Q value.

### Interpretation

To the best of our knowledge, our study is the first to quantify the difference in the extent of prolapse between POP-Q and upright MRI measurements. The underestimation of POP by POP-Q compared with MRI-PICS can be explained by several arguments. Most important is the patient position during the examination. The POP-Q measurements are performed in a dorsal lithotomy position during Valsalva maneuver, whereas the MRI-PICS measurements are performed in an upright position with the pelvis at rest. Previous studies already concluded that the supine straining position can lead to an underestimation of POP, as not all patients are able to perform Valsalva correctly [18]. The underestimation of uterus prolapse we determined is in line with earlier research that compared POP-Q measurement with Valsalva with POP-Q measurement under traction intra-operatively. An increase in uterus prolapse in up to 93% of patients was found in these studies [8–10].

The implications of this study for the individual patient are of utmost importance because underestimation of the degree of POP can lead to under-treatment. For instance, if a patient suffers from POP symptoms, but no prolapse is seen on physical examination, no treatment is recommended, and the patient continues to suffer the same complaints. Additionally, in planning POP surgery, underestimation of POP can lead to choosing the wrong or incomplete technique for correcting POP. This may lead to early recurrence of POP and the need to perform recurrence surgery. Considering this, ideally, we recommend performing upright MRI-PICS measurements in all patients with POP for whom the symptoms are worse than expected based on the extent of POP seen on physical examination.

Apart from the correlation between MRI-PICS measurements and POP-Q measurements, future research should focus on the correlation between MRI-PICS measurements and symptoms. Previous research showed that symptoms correlate poorly with POP-Q stage, which might now be explained by the underestimation of POP. In that case MRI-PICS measurements could show a better correlation with complaints.

## Conclusion

In this prospective study we showed that despite a moderate positive correlation between the POP-Q measurements and the upright MRI-PICS measurements in patients with POP, an underestimation of POP when assessed using POP-Q is found. The great variation in MRI-PICS measurement in patients with similar POP-Q values supports our hypothesis that upright MRI might give a more reliable estimation of the extent of prolapse of the bladder and uterus. We recommend further research to consider the correlation between POP symptoms and MRI-PICS measurements, to conclude whether upright MRI is of value for patients in whom symptoms are worse than expected based on POP-Q.
